# Liposomal amphotericin B in travelers with cutaneous and muco-cutaneous leishmaniasis: Not a panacea

**DOI:** 10.1371/journal.pntd.0006094

**Published:** 2017-11-20

**Authors:** Romain Guery, Benoit Henry, Guillaume Martin-Blondel, Claire Rouzaud, Florence Cordoliani, Gundel Harms, Jean-Pierre Gangneux, Françoise Foulet, Emmanuelle Bourrat, Michel Baccard, Gloria Morizot, Paul-Henri Consigny, Antoine Berry, Johannes Blum, Olivier Lortholary, Pierre Buffet, the French Cutaneous Leishmaniasis Study group & the LeishMan network

**Affiliations:** 1 Service de Maladies Infectieuses et Tropicales, Centre d’Infectiologie Necker-Pasteur, Assistance Publique-Hôpitaux de Paris, Hôpital Necker Enfants Malades, Université Paris Descartes, Sorbonne Paris Cité, Paris, France; 2 Service des Maladies Infectieuses et Tropicales, CHU de Toulouse, INSERM U1043—CNRS UMR 5282, Centre de Physiopathologie Toulouse-Purpan, Toulouse, France; 3 Service de Dermatologie, Hôpital Saint-Louis, Assistance Publique-Hôpitaux de Paris, Université Paris Diderot-Paris VII, Paris, France; 4 Institute of Tropical Medicine and International Health, Charité—Universitätsmedizin Berlin, Berlin, Germany; 5 Service de Parasitologie-Mycologie, CHU de Rennes, INSERM U1085, Université Rennes 1, Rennes, France; 6 Unité de Mycologie, Département de Biologie-Pathologie, CHU Henri Mondor, DHU VIC, Assistance Publique-Hôpitaux de Paris, Créteil, France; 7 Service de Pédiatrie Général, Hôpital Robert Debré, Service de Dermatologie, Hôpital Saint-Louis, Assistance Publique-Hôpitaux de Paris, Université Paris Diderot-Paris VII, Paris, France; 8 Plateforme ICAReB, Institut Pasteur, Paris, France; 9 Centre Médical de l'Institut Pasteur, Consultation de Maladies Infectieuses, Tropicales et de Médecine des Voyages, Centre d'Infectiologie Necker-Pasteur, Institut Pasteur, Paris, France; 10 Service de Parasitologie-Mycologie, CHU de Toulouse, INSERM U1043—CNRS UMR 5282, Centre de Physiopathologie Toulouse-Purpan, Toulouse, France; 11 Swiss Tropical and Public Health Institute, University of Basel, Basel, Switzerland; 12 INTS, Unité Biologie Intégrée du Globule Rouge, Laboratoire d'Excellence GR-Ex, Centre d'Infectiologie Necker-Pasteur, Institut Pasteur, Paris, France; National Institutes of Health, UNITED STATES

## Abstract

**Background:**

Complex cutaneous and muco-cutaneous leishmaniasis (CL and MCL) often requires systemic therapy. Liposomal amphotericin B (L-AmB) has a strong potential for a solid clinical benefit in this indication.

**Methods:**

We conducted a retrospective analysis of data from a French centralized referral treatment program and from the “LeishMan” European consortium database. All patients with parasitologically proven CL or MCL who received at least one dose of L-AmB were included. Positive outcome was based on ulcer closure as per recent WHO workshop guidelines.

**Results:**

From 2008 through 2016, 43 travelers returning from 18 countries (Old World n = 28; New World n = 15) were analyzed with a median follow-up duration of 79 days [range 28–803]. Main clinical forms were: localized CL with one or multiple lesions (n = 32; 74%) and MCL (n = 8; 19%). As per published criteria 19 of 41 patients (46%) were cured 90 days after one course of L-AmB. When the following items -improvement before day 90 but no subsequent follow-up, delayed healing (>3 months) and healing after a second course of L-AmB- were included in the definition of cure, 27 of 43 patients (63%) had a positive outcome. Five patients (MCL = 1; CL = 4) experienced a relapse after a median duration of 6 months [range 3–27] post treatment and 53% of patients (23/43) experienced at least one adverse event including severe hypokalaemia and acute cardiac failure (one patient each). In multivariate analysis, tegumentary infection with *L*. *infantum* was associated with complete healing after L-AmB therapy (OR 5.8 IC 95% [1.03–32]) while infection with other species had no impact on outcome.

**Conclusion:**

In conditions close to current medical practice, the therapeutic window of L-AmB was narrow in travellers with CL or MCL, with the possible exception of those infected with *L*. *infantum*. Strict follow-up is warranted when using L-AmB in patients with mild disease.

## Author summary

Cutaneous and muco-cutaneous leishmaniasis (CL/MCL) are disfiguring diseases caused by a worldwide distributed parasite called *Leishmania* and its 20 species. Clinical manifestations span a wide continuum from single nodular lesion to disseminated form with mucosal involvement. Though local treatment with cryotherapy and intralesionnal antimony or topical formulations of paromomycin is generally adequate in most of situations, some patients with complex CL/MCL require systemic therapy. No convenient regimen has been proved to be safe and effective for all infecting species, all clinical forms and all patients (e.g. children, pregnant women, adults with comorbidities or immunosuppression). In this study, the authors examined in returning travelers with CL/MCL the effectiveness of an antifungal agent “liposomal amphotericin B” (L-AmB), which is highly effective in visceral leishmaniasis. Surprisingly, rates of healing were lower than in previous reports in this unselected population that reflects clinical practice in non-endemic countries. The observations also suggest that some *Leishmania* species (namely, *L*. *infantum*) may be more susceptible to L-AmB than others. Occurrence of adverse events should raises the question of the benefit-risk balance of L-AmB in CL/MCL. Careful attention to comorbidities and adoption of strict protocols for administration are pre-requisites for the use of L-AmB in patients with CL/MCL.

## Introduction

Tegumentary leishmaniasis (TL) occurs when one of the 20 *Leishmania* species that can infect humans causes lesions of the skin or mucosae. Because TL is observed in patients of all ages, either immunocompetent or immunocompromised, the result is a wide continuum of clinical forms and medical situations for which a multiplicity of treatment approaches have been proposed. Despite the high global burden of cutaneous and muco-cutaneous leishmaniasis (CL and MCL) and the rising number of travels to endemic areas, there is still relatively sparse evidence to support an accurate choice between local (e.g. cryotherapy and/or intralesional antimony, topical paromomycin) or systemic therapy (e.g. amphotericin B, miltefosine, pentavalent antimonials, pentamidine, fluconazole). In addition, as almost all studies have been conducted in endemic countries, published research may not apply to travelers [1]. National and international recommendations as well as recent IDSA guidelines favor systemic treatment for patients with complex CL defined by a high risk of mucosal involvement (if this can be accurately determined), numerous or very large lesions, disseminated forms, lesions location not compatible with local treatment (e.g. periorificial, fingers, toes, ears), or immunosuppression [1–4].

Among the currently available systemic antileishmanial agents, liposomal amphotericin B (L-AmB) has a strong potential for a high clinical benefit in TL. L-AmB has a pivotal role in the management of visceral leishmaniasis (VL) as efficacy of 95 to 100% has been reported both in the Indian subcontinent (where VL is due to *L*. *donovani*) and in Southern Europe (where VL is due to *L*. *infantum)* [5–7]. The drug has been used off-label for imported CL in various settings and response rates of 75 to 85% have been reported in travelers who had been predominantly infected in the New-World [8,9]. Cure rates around 90% were reported in the treatment of MCL with deoxycholate amphotericin B or with liposomal forms [10]. Due to its extensive use in fungal infections and in VL, the safety profile of L-AmB is well-known as well as how to prevent and manage its renal and metabolic adverse events.

Nevertheless—possibly because of the high cost of L-AmB—relatively little information has been reported so far on the clinical benefit of L-AmB in CL in general and in Old-World CL in particular. Less than 50 patients infected in the Old World (including children) were treated in the three largest published studies [8,9,11]. Because these studies included predominantly patients without comorbidities, immunosuppression or complex CL, information on rare side effects affecting fragile patients could not be captured.

The objective of this retrospective study was to assess the efficacy and safety of L-AmB for the treatment of TL in unselected travelers of all age groups returning from both the Old or New World.

## Methods

### Ethics statements

French National Agency regulating data protection (Commission Nationale de l’Informatique et des Libertés) approved this observational study (DR-2013-041; N°912650). All data were recorded anonymously through the approved database. As diagnosis and treatment strategy followed the international or national guidelines (of each country) there is no ethical concern on treatment and diagnosis. Patients (or parents or legal guardians of children) gave their informed oral or written consent allowing examinations on their samples and publication of anonymized data on clinical findings, treatment received, clinical outcome and laboratory examinations.

### Data collection

From 2008 through 2012 data were collected in a French nationwide centralized referral treatment program as reported elsewhere [12]. Briefly, these data were collected by experts providing treatment advice to physicians attending patients with leishmaniasis in France. Expert advice was part of normal medical process and followed the national guidelines [2]. Demographic and clinical data were obtained from treating physicians through a standardized case report form. After at least 45 days after the treatment advice has been provided, the attending physician was contacted and asked to provide follow-up information, using a standardized form, regarding the identification of the infecting *Leishmania* species, treatment currently administered, treatment outcome, and adverse events.

From 2012 through 2016, data were collected in the European LeishMan (“Leishmaniasis management”) database, a multicentre multinational surveillance project on cutaneous and mucosal leishmaniasis. The consortium gathers 17 experts from 12 institutions in 7 European countries (France, Switzerland, United-Kingdom, Belgium, Netherlands, Germany and Spain) who aim at improving the management of leishmaniasis through harmonization of medical practices and collection of data in an international surveillance system [13]. Demographic, clinical and biological data were assessed in an electronic case report form and documented before and after treatment. Treatment schedule was left at the discretion of the attending physician, unless he/she explicitly required advice on this point from the expert.

### Patients

All patients with parasitologically confirmed cutaneous or mucosal leishmaniasis who received at least one infusion of liposomal AmB (Ambisome^®^) were included. Only patients with subsequent follow-up were included in the final analysis.

### Diagnostic methods

Parasitological diagnosis was based on: visualization of amastigotes on stained smear obtained by scrapping or histological tissue sections and/or promastigotes in culture from material obtained by aspiration and/or positive polymerase chain reaction (PCR) on material obtained by aspiration or by skin biopsy. Whenever possible, samples and aliquots of positive cultures were sent to the French National Reference Center Leishmaniasis for species identification using a multilocus sequence typing approach based on the analysis of seven single-copy coding DNA sequences or using MALDI-TOF mass spectrometry as reported elsewhere [14].

### Outcome

Success was defined as complete healing within 3 months after starting therapy. Healing was assessed either by closure of the ulceration (if present) or disappearance of the induration if no ulceration was present. Failure was defined as incomplete reepithelialization or persistence of erythematous induration at day 90. Improvement corresponds to partial response before evaluation at day 90 and no subsequent follow-up. A relapse was defined as reappearance of cutaneous or mucosal lesions after complete healing without evidence of reinfection during follow-up. All events, whether related or not to L-AmB therapy following treatment administration were considered as adverse events. Adverse events were considered severe if life threatening.

### Data analysis

Continuous variable are presented as median [range] and categorical variables as numbers (frequencies). Categorical variables were compared using chi-square test or Fisher’s exact test as appropriate. Differences in mean values between two groups were compared using Mann Whitney test. A two-tailed p-value < .05 was considered as statistically significant. Univariate analysis was followed by multivariable logistic regression analysis to define predictive factors of complete healing after L-AmB therapy. All statistical analyses were performed using StatView software (Version 5.0 SAS institute Inc., Cary, NC).

## Results

### Characteristics of patients

From 2008 through 2016, data from 45 patients who had received at least one infusion of L-AmB for TL were entered into the databases. Two patients had no-follow-up and were excluded from the analysis. The characteristics of 43 travelers returning from 18 different countries are shown in **Table 1**. Twenty nine centers from 3 countries (26 in France, 2 in Switzerland, 1 in Germany) participated to the study. Leishmaniasis was mainly acquired in the Old World (9 in Europe, 9 in Maghreb, 6 in sub-Saharan Africa, 3 in Middle East, 1 in India). 15 patients were infected in the New World (10 in French Guiana, 3 in Bolivia, 1 in Brazil, 1 in Peru). Median age of the cohort (which comprised 6 children aged from 1 to 12 years) was 55 years for adults. Seven patients (19% of adults) had prior cardiovascular history. Among immunocompromised patients (5/43; 12%), three had human immunodeficiency infection (HIV) infection and two received prolonged corticosteroid therapy and/or immunosuppressive treatment for autoimmune diseases.

**Table 1:** Main characteristics of 43 patients with tegumentary leishmaniasis treated with liposomal amphotericin B.

Characteristics of patients		n = 43
**Demographics**		
	Median age, years [range]	51 [1–86]
	Male	29 (67)
	Cardiovascular comorbidities and/or diabetes	7 (16)
	Immunocompromised subject	5 (12)
	Child	6 (14)
**Clinical form**		
	Localized cutaneous	32 (74)
	Mucocutaneous	8 (19)
	Disseminated cutaneous	2 (5)
	Localized cutaneous with visceral involvement	1 (2)
**Area where infection was acquired**		
	Old World	28 (65)
	New World	15 (35)
***Leishmania* Species (n = 35; 8 species unidentified)**		
	*L*. *braziliensis*	11 (31)
	*L*. *braziliensis complex*	2 (6)
	*L*. *guyanensis*	1 (3)
	*L*. *amazonensis*	1 (3)
	*L*. *infantum*	9 (26)
	*L*. *major*	6 (17)
	*L*. *tropica*	3 (8)
	*L*. *donovani*	2 (6)
**Clinical findings**		
	Number of lesions, median [range]	2 [1–30]
	Larger lesion size (millimeter), median [range]	30 [4–200]
**Treatment**		
	Frontline therapy with L-AmB	30 (70)
	Liposomal AmB cumulative dose (mg/kg), median [range]	20 [6–56]
	Number of infusions, median [range]^a^	6 [2–14]
**Outcome**		
	Follow-up (days), median [range]	79 [28–803]
	Complete healing without relapse	19 (44)
	Improvement	2 (5)
	Failure	17 (39)
	Relapse	5 (12)
**Adverse events**		
	Patients with at least one adverse event	23 (53)
	Treatment modification due to adverse events	7 (16)

L-AmB, liposomal amphotericin B. Data are n (%) unless otherwise indicated.

^a^ this information was available for 24 patients

### Clinical and biological findings

Localized CL (n = 32; 74%) was the predominant clinical form, followed by muco-cutaneous leishmaniasis (n = 8; 19%). One subject with HIV infection had CL with simultaneous visceral involvement caused by *L*. *donovani*, and two other patients had disseminated form (*L*. *major*; *L*. *guyanensis*) defined as more than 10 skin lesions in two non-contiguous sites. 72% of patients had at least two lesions and 28% (12/43) had at least one large lesion (≥ 40mm). Among 32 patients with CL, 10 (31%) had lesions on the face. Median duration of disease was 5 months [range 1–24] but this information was available for only 15 patients. The infecting *Leishmania* species was identified in 35 patients (81%). 14 species belonged to the *Viannia* subgenus (40%), the other common species were *L*. *infantum* (n = 9; 26%) and *L*. *major* (n = 6; 17%).

### Treatment

Median cumulative dose of L-AmB per patient was 20 mg/kg [range 6–56]. Most of patients were treated with the “VL” regimen (i.e., daily infusion from day 1 to day 5 followed by one infusion on day 10) but data on duration of treatment were missing for 19 patients. Two third of patients (30/43; 70%) received L-AmB as front-line therapy while the others had received prior treatment for leishmaniasis with pentavalent antimony (n = 6), pentamidine (n = 4), oral azoles (n = 3) or local treatment (n = 3). Three patients received concomitant medication before day 90, with fluconazole (n = 1) or miltefosine (n = 2) during L-AmB therapy due to premature discontinuation of L-AmB for adverse events.

### Adverse events

No death was reported. Renal toxicity and infusion related reactions occurred in 15 (35%) and 6 (14%) patients respectively. A 81 year-old patient with hypertensive heart disease experienced acute heart failure after hydratation with saline solution during L-Amb therapy. One week after the last infusion a 29 year-old patient developed severe symptomatic hypokaliemia (2.5mmol/L) without ECG changes which required hospitalization and intravenous potassium chloride supplementation. While 53% of patients (23/43) experienced at least one adverse event, seven of them (30%) required modification of L-AmB therapy during follow-up (discontinuation n = 3; dose delay n = 3; dose reduction n = 1).

### Outcome

Median follow-up was 79 [28–803] days. L-AmB therapy was associated with a complete healing (without relapse) or improvement before day 90 in 21 patients (49%) **(Table 1)**. Five patients experienced a relapse after a median of 6 months [range 3–27] post treatment. Of the five patients who relapsed, four (80%) were infected by a *Leishmania* species belonging to the *Viannia* subgenus. Of the 22 patients with a negative outcome before day 90, 3 (14%) had delayed healing after 5 to 18 months, while 3 (14%) healed after a second course (new cure with 20mg/kg cumulative dose) of L-AmB, 2 (9%) with pentavalent antimony and 2 (9%) with miltefosine. If delayed healing and healing after a second course of L-AmB are considered positive outcomes the healing rate in L-AmB treated patients was 63%. **Table 2** provides effectiveness analysis in relation to subgroups of patients.

**Table 2:** Predictors of success of liposomal amphotericin B in 41 patients with tegumentary leishmaniasis.

			Complete healing without relapse	Failure or relapse	Success of L-AmB	p value
			n = 19^a^	n = 22^a^	(%)	
**Patients**						
	Age		57 [2–80]	42 [1–86]	/	0.58
	Immunocompromised subject		2 (10)	3 (14)	40	0.99
	Localized cutaneous form		14 (74)	17 (77)	45	0.99
	Mucocutaneous form		4 (21)	3 (14)	57	0.68
	Frontline therapy with L-AmB		15 (79)	15 (68)	50	0.44
**Country where infection was acquired**						
	Old World		15 (79)	11 (50)	58	0.05
	New World		4 (21)	11 (50)	27	0.05
**Leishmania species**						
	*Viannia* subgenus		4 (21)	10 (45)	28	0.13
	*L*. *infantum*		7 (37)	2 (9)	78	0.06
	*L*. *major*		2 (10)	4 (18)	50	0.99
**Clinical findings**						
	Number of lesions		2 [1–30]	2 [1–8]	/	0.84
**Treatment**						
	Cumulative dose of L-AmB (mg/kg)		20 [6–40]	20 [16–56]	/	0.73

L-AmB, liposomal amphotericin B. Data are represented as n (%) or median [range] unless otherwise indicated.

^a^Two patients with improvement at first control visit (before day 90) but no subsequent follow-up were excluded from the analysis.

### Predictors of L-AmB success

In univariate analysis (**Table 2)**, the area where infection was acquired was associated with L-AmB effectiveness (p≤ .05). Being infected with *L*. *infantum* approached but did not quite achieve significance (p = .057). No other clinical or biological factors were associated with the outcome after L-AmB therapy. Of the 9 patients with *L*. *infantum* tegumentary leishmaniasis, 5 (55%) had mucocutaneous and 4 (45%) localized cutaneous leishmaniasis. The median number of lesions was 2 [range 1–17]. There were two failures: one mucocutaneous form of the lips and one cutaneous form of the leg. The following variables *L*. *infantum* species, area (New World or Old World) where infection was acquired, mucocutaneous form, and frontline therapy with L-AmB were included in multivariable conditional logistic regression analysis. In multivariable analysis, only infection with *L*. *infantum* was independently associated with complete healing after L-AmB therapy (OR 5.8 IC 95% [1.03–32]).

## Discussion

In this cohort of unselected travellers with TL, L-AmB was associated with a suboptimal clinical benefit. Following conventional criteria for healing (excluding patients with improvement before day 90 and no subsequent follow-up), only 19 of 41 patients (46%) were cured 90 days after one course of L-AmB and when the most inclusive estimate was used—which integrates improvement before day 90 and no subsequent follow-up, delayed healing (>3 months) and healing after a second course of L-AmB—the cure rate reached a still relatively low 63% cure rate (27 of 43 patients) [15]. We also observed clinically significant adverse events. Renal toxicity (including electrolytic disorders and acute renal failure) and infusion related events (fever, chills) occurred in 35% and 14% of patients respectively. In 7 of 43 patients, discontinuation or modification of the initially prescribed regimen of L-AmB was deemed necessary by attending physicians. Two patients experienced serious adverse events. Acute cardiac failure occurred in an 81 year-old patient with a pre-existing cardiovascular condition, and delayed severe hypokaliemia occurred in a 29 year-old patient without pre-existing condition. In current medical practice, the risk-to-benefit ratio of L-AmB may thus be lower in TL than in VL [5,7]. A puzzling question is whether the relatively high risk of severe adverse events observed in this cohort was counterbalanced by the (inconstantly) positive impact of treatment, especially in patients with disfiguring but not life-threatening cutaneous lesions.

Why would efficacy of liposomal amphotericin B be lower in TL than in VL? To our knowledge, no study has systematically evaluated skin penetration of L-AmB following systemic administration in human subjects. Penetration of amphotericin B was lower in the skin than in other organs in a rat model suggesting that higher doses may be needed in TL as compared to VL, although this will likely increase toxicity and inevitably increase cost [16]. Predictions are elusive due to the non-linear pharmacokinetics and complex mechanisms of reticuloendothelial uptake and release of liposomal amphotericin B, likely influenced by the presence of skin lesions [17,18]. Parasite “resistance” to L-AmB in TL may be another important parameter to explain poor efficacy in this context. Although substantiated so far only by circumstantial evidence, a dormant metabolic state of parasites in skin lesions may also contribute to the suboptimal effectiveness of L-AmB in CL, a process suspected to occur only rarely in VL [19–21]. A species-related difference in drug susceptibility is a third potentially important determinant of outcome in TL and VL. That efficacy was high when TL was due to *L*. *infantum* (approximately 80%) and low when it was due to other identified species (around 30%) provides some substance to this assumption. Of note, variations of susceptibility to L-AmB may even exist at the intra-species level as VL due to *L*. *donovani* is less responsive to L-AmB in East Africa than it is in India [22]. Apart from the impact of *L*. *infantum*, we found no obvious epidemiological, clinical or laboratory parameter associated with outcome after treatment with L-AmB in TL. L-AmB has been successfully used in multiple case reports where TL was caused by *L*. *infantum* but whether TL due to *L*. *infantum* is indeed more sensitive to L-AmB—as our multivariate analysis suggests—will requires assessment in larger studies [23,24]. In this study, we pooled CL and MCL which increased the power of the analysis to identify potential predictors of outcome. We observed no significant difference in efficacy in patients with or without mucosal involvement which provides some post-hoc justification to this approach. Multinational networks like LeishMan, will help increase the statistical power to determine the potential impact of host characteristics, clinical forms and infecting species on the outcome of each form analyzed separately.

Healing rates were also lower in our cohort than in previous studies that had assessed the efficacy of L-AmB in TL, somewhat reminiscent of the decline in efficacy observed across the evaluation process with L-AmB and miltefosine in VL and with miltefosine or azoles in TL [25–27]. We observed a 27% of efficacy of L-AmB in TL acquired in the New World while it had varied from 80% to 100% in previous studies, except in one where low (7.5 mg/kg) cumulative dose of L-AmB had been used [9,25–29]. To our knowledge, standard regimens of L-AmB have not been comparatively evaluated in TL and so far, randomized controlled studies with this drug have been performed only in VL. In TL, most observations report on a cumulative dose of 20mg/kg, likely by extrapolation from guidelines for VL. This regimen is similar to the median dose used in our study. It has been suggested that a higher cumulative dose (>30mg/kg) could be beneficial in disseminated forms caused by *L*. *braziliensis* [30]. Toxicity and cost are likely to increase with the cumulative dose used. Regarding patients who acquired TL the Old World, we observed a 58% cure rate while it was 75% and 84% in the two largest studies published so far [8,11]. These studies involved patients infected with *L*. *tropica*, in keeping with the observation that the three patients infected with *L*. *tropica* in our study had also a positive outcome. As mentioned above regarding *L*. *infantum*, the infecting species may thus influence the efficacy of L-AmB in TL although others factors such as age, presence of immunosuppression or comorbidities may have also affected the results. The population was diverse in our study which included subjects visiting friends and relatives, expatriates, military personal and tourists while previous studies had included very predominantly young military personal or children. Not least, part of the relatively low cure rate in our study may result from the frequent discontinuation or modification of L-AmB regimen.

In summary, while L-AmB may not have been given its best chance of success in our study its medical impact was assessed in conditions close to that prevailing in current medical practice. The therapeutic window of L-AmB, indisputably wider than that of other antileishmanial drugs in VL, appeared unexpectedly narrow in this cohort of unselected travellers with TL, with the possible exception of TL due to *L*. *infantum*. The momentum should be maintained to deliver either optimized regimens of existing antileishmanial drugs in TL (either systemic or local), or new drugs which should ideally be amenable to oral administration [31]. Such a perfect drug does not exist at the moment. In the short term, physicians should pay close attention to the potential side effects of L-AmB related to comorbidities and adopt strict clinical and laboratory follow-up when using L-AmB in patients with TL. The risk to benefit ratio of L-AmB in patients with mild CL may be difficult to determine which brings further support to the use of local therapy as front-line approach, as now recommended [2,32]. Beside cryotherapy plus intralesional antimony, topical paromomycin has an excellent risk-benefit ratio but is still not available due to unexplained delays in its clinical development despite positive clinical trials [33–35].

In travelers with complex CL or MCL not due to *L*. *infantum*, oral miltefosine, pentavalent antimony, pentamidine and L-AmB should be considered as front-line therapy with the choice guided by age of the patient and pre-existing comorbidities.

## Supporting information

S1 Checklist STROBE checklist. (DOC)
